# High-resolution combinatorial patterning of functional nanoparticles

**DOI:** 10.1038/s41467-020-19771-0

**Published:** 2020-11-26

**Authors:** Xing Xing, Zaiqin Man, Jie Bian, Yadong Yin, Weihua Zhang, Zhenda Lu

**Affiliations:** 1 College of Engineering and Applied Sciences, State Key Laboratory of Analytical Chemistry for Life Science, and Jiangsu Key Laboratory of Artificial Functional Materials, Nanjing University, Nanjing, 210093 PR China; 2 Department of Chemistry, University of California, Riverside, CA, 92521 USA; 3 MOE Key laboratory of Intelligent Optical Sensing and Manipulation, Nanjing University, Nanjing, 210023 PR China; 4 Research Center for Environmental Nanotechnology (ReCENT), Nanjing University, Nanjing, 210023 PR China

**Keywords:** Surface assembly, Surface patterning, Nanoscience and technology

## Abstract

Fast, low-cost, reliable, and multi-component nanopatterning techniques for functional colloidal nanoparticles have been dreamed about by scientists and engineers for decades. Although countless efforts have been made, it is still a daunting challenge to organize different nanocomponents into a predefined structure with nanometer precision over the millimeter and even larger scale. To meet the challenge, we report a nanoprinting technique that can print various functional colloidal nanoparticles into arbitrarily defined patterns with a 200 nm (or smaller) pitch (>125,000 DPI), 30 nm (or larger) pixel size/linewidth, 10 nm position accuracy and 50 nm overlay precision. The nanopatterning technique combines dielectrophoretic enrichment and deep surface-energy modulation and therefore features high efficiency and robustness. It can form nanostructures over the millimeter-scale by simply spinning, brushing or dip coating colloidal nanoink onto a substrate with minimum error (error ratio < 2 × 10^−6^). This technique provides a powerful yet simple construction tool for large-scale positioning and integration of multiple functional nanoparticles toward next-generation optoelectronic and biomedical devices.

## Abstract (web summary)

Precise patterning of functional nanoparticles can provide a powerful tool for next-generation macroscale devices. Here, the authors report a reliable nanoprinting technique that can pattern various functional nanoparticles on the substrate with a 200 nm pitch and 10 nm position accuracy, and above the millimeter scale.

## Introduction

Nanofabrication, the process of making miniature structures with nanometer precision, is the foundation of integrated electronics that has profound impacts on our modern society. With advanced photolithographic tools, people can now produce transistors with feature sizes below 10 nm over 12 inch Si wafers at high yields. However, when it comes to the emerging areas, such as photonic, optoelectronic, and biomedical nanodevices, where materials are often incompatible with the conventional CMOS processes, more nanofabrication tools are highly desirable. One possible solution is the additive fabrication through wet chemical self-assembly, which can organize pre-synthesized colloidal nanoparticles (NPs) as functional building blocks at the desired positions to build devices from bottom up^1–3^. With the recent significant advances in colloidal synthesis, NPs of metal, semiconductor, and dielectrics with unique electrical, magnetic, and optical properties can now be produced routinely in large quantity and high quality^4,5^. However, until now it still remains a great challenge in finding robust assembly approaches to effectively introduce NPs of different compositions and functions to the pre-designed locations^6,7^, although several methods have been proposed in the past two decades to tackle this issue, including colloidal self-assembly^8,9^, microcontact printing^10,11^, dip-pen nanolithography^12^, inkjet printing^13^, optical tweezers^14^, biomolecule-induced assembly^15^, patterning within predefined features^16^ or chemically modified surface^17^, nanoxerography^18–23^, and so on. These previous methods are not yet ready for broad practical applications as they suffer from various limitations such as small scale, slowness, low resolution, and inflexibility with the choice of NPs and substrates. Techniques for robust, fast, multi-component NP patterning with true nanometer precision and high scalability are still highly demanded, which, if made available, are believed to revolutionize the manufacturing processes toward many advanced nanodevices^7,24^.

The difficulty of achieving robust nanopatterning of functional NPs is rooted in the complexity of the NPs behaviors at the solution/substrate interface^25^. To align NPs at defined locations with nanometer precision, it requires a series of interactions between NPs and substrate. It needs long-range particle-substrate attraction to enrich the NPs on the substrate in order to realize efficient and fast assembly, strong short-range attraction to fix the NPs at the designed locations, and counter-interactions to avoid nonspecific deposition of NPs at undesired areas. Moreover, all these interactions are sensitive to the subtle changes of assembly parameters, such as the surface property of the substrate, solvent polarity, and particle morphology, and they are often coupled and affecting each other. A robust nanoprinting technique will need to carefully optimize these interactions to enable effective NP assembly.

In this work, we report an electric field-assisted surface-sorption nano-printing (EFASP) method for patterning multiple functional nanoparticles with high resolution, precision, and scalability. As an additive nanofabrication method, it utilizes a tip-based high-voltage writing process to generate nanoscale de-fluorinated patterns in a fluorinated surface, which not only enables electrostatic trapping but also creates a high local surface potential in contrast to the fluorinated area, both favoring highly efficient site-specific NP assembly. By combining long-range electrostatic attractions in the solvent, short-range surface adsorption, and low surface energy of the fluoride substrate, EFASP allows fast, nanometer precision printing of various functional NPs over large areas with high reproducibility. The advantages of EFASP also include combinatorial patterning of multiple functional nanoparticles for realizing multi-color printing by conveniently repeating the writing and assembly cycles.

## Results

### Electric field-assisted patterning of NPs

**Fig. 1:**
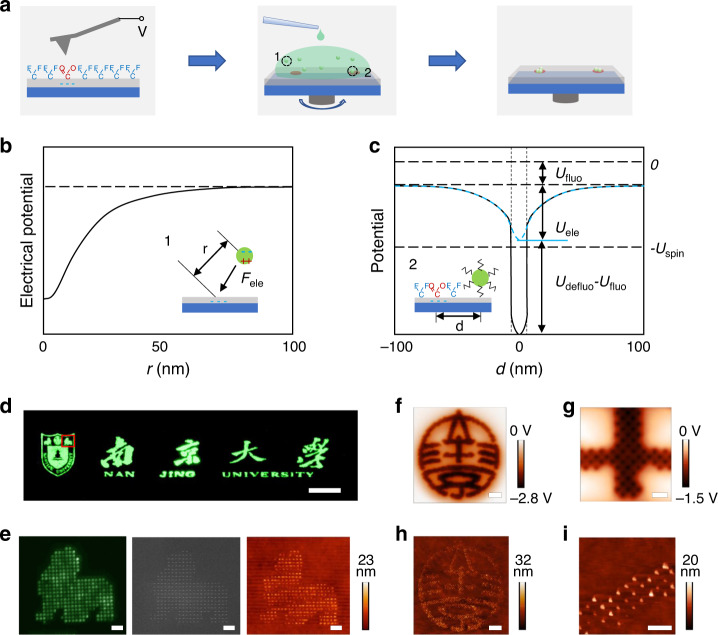
Nanoprinting of perovskite QDs by EFASP. **a** Schematic illustration of the EFASP process, which involves high-voltage electrostatic nanopattern generation, surface modification, and site-specific NP assembly by electrical trapping. **b** Electrical trapping potential in a nonpolar solvent as a function of the distance from the center of the written charge (dash circle 1 in **a**). The electric fields can extend for hundreds of nanometers, leading to the high efficiency of NP collection. **c** Surface potential modulation along the nanopattern surface (dash circle 2 in **a**) De-fluorination through high voltage induces significant surface energy modulation, providing strong short-range interactions to fix the NPs. **d** A large-scale photoluminescence (PL) image obtained by assembling CsPbBr_3_ NPs using the EFASP process, with a total area of 100 × 800 μm^2^ with 9481 pixels, a pitch of 800 nm, and approximately 4.7 × 10^5^ 10-nm CsPbBr_3_ NPs. Scale bar, 50 μm. **e** High-magnification PL, SEM, and AFM height images of the marked area in **d**. Scale bar, 2 μm. **f**–**i** Extremely high-resolution EFASP can be produced by modulating patterns, reaching a pitch of 200 nm (125,000 DPI). The high-magnification KPFM potential map and corresponding AFM height map after NP assembly (**f** and **h**, scale bar: 2 μm) and their details (**g** and **i**, scale bar: 500 nm). The corresponding large area PL image is shown in Supplementary Fig. 2.

The EFASP process is schematically illustrated in Fig. 1a. A charged nanopattern is first written on a fluorine polymer substrate (electret) by applying a localized high voltage (-40 to -90 V) using a conductive atomic force microscope (AFM) tip. Here, the localized high voltage decomposes the fluorine polymer surface with nanoscale precision and creates de-fluorinated nanopatterns, which can be used for effectively attracting colloidal nanoparticles (Fig. 1b, c). Then, an oil dispersion of NPs is applied to the substrate. NPs in the nonpolar solvent is enriched at the areas of electrostatic nanopatterns due to the electrostatic attraction and adsorbed on the patterned areas because of their high surface energy. In the unpatterned area, no adsorption occurs since the surface energy of the fluoride substrate is low and all NPs are carried away by the solution.

With this method, we can accurately assemble perovskite quantum dots (QDs) in an area of 100 × 800 μm^2^, as shown in the photoluminescence (PL) image of Nanjing University Logo, composed of 9481 pixels, approximately 4.7 × 10^5^ 10-nm CsPbBr_3_ NPs (Fig. 1d). No nonspecific adsorption was found in the uncharged area, as shown in the enlarged PL, SEM, AFM images in Fig. 1e. In other words, the error ratio (i.e., the ratio of the number between nonspecific adsorbed and correctly printed NPs) is lower than 2 × 10^-6^. This extremely low error ratio ensures the EFASP a reliable nanofabrication technique. Moreover, EFASP enjoys a very high resolution and accuracy. The pitch size can reach 200 nm (125,000 DPI), or even a smaller value, as supported by the Kelvin probe force microscopy (KPFM) potential maps (Fig. 1f, g) and the corresponding AFM height maps of the printed structure (Fig. 1h, i), with the positioning accuracy better than 10 nm (Supplementary Fig. 1). The nanoprinting method is robust and fast, which can be completed by not only spin coating but also brushing or dip coating of the NP dispersion in just seconds. (Supplementary Movie 1)

### Combinatorial patterning

**Fig. 2:**
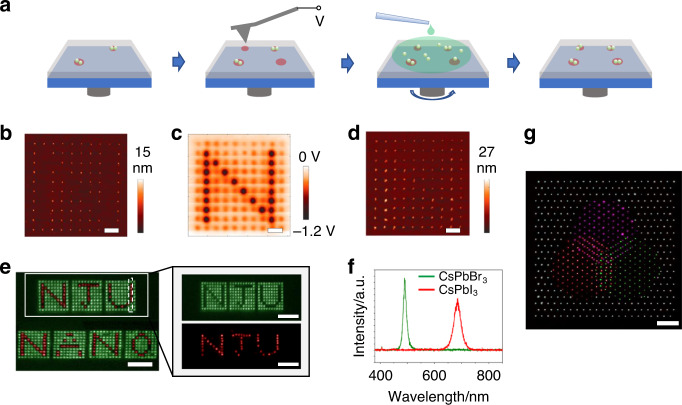
Color printing of multiple functional NPs. **a** Schematic illustration of the assembly of different NPs by repeating the EFASP process. **b** AFM height image after printing the first cycle of 12-nm CsPbBr_3_ QDs as a hollow N pattern. Scale bar, 1 μm. **c** Electrical potential image after writing the second charge pattern N. Scale bar, 1 μm. **d** AFM height mapping of the binary pattern after depositing the second cycle of CsPbI_3_ QDs. Scale bar, 1 μm. **e** PL image of the dual-color pattern and its corresponding images with green and red bandpass filters (marked by the white solid rectangle). No spatial overlap was found between the green and red areas, indicating no cross-contamination occurs. Scale bar, 5 μm. **f** PL spectra of two kinds of QDs on one pixel corresponding to (**e**). **g** pseudo-color image captured by the dark-field microscopy of a color pattern, which is composed of four types of NPs with 606 pixels. The white color is created by 15-nm γ-Fe_2_O_3_ NPs, while the magenta, green and red colors are produced by NaYF_4_:Yb,Er, CsPbBr_3_ and CdSe@ZnS NPs, respectively. Scale bar, 10 μm.

The EFASP technique can be directly extended to color patterning by repeating the writing/deposition cycles. Here, we used a dual-color pattern with two different NPs, green CsPbBr_3_ and red CsPbI_3_ QDs as an example to illustrate the process (Fig. 2a). First, CsPbBr_3_ QDs were printed, forming a hollow NJU pattern. Then, we scanned the as-formed pattern with AFM, aligned the probe using the topography image as the reference, and wrote a new pattern for the second NP deposition cycle (Fig. 2b, c). This process guarantees the overlay accuracy between different printing cycles, and in our work, it reaches 58 nm (Supplementary Fig. 3). After that, as shown in Fig. 2d we deposited CsPbI_3_ QDs by spin coating, and complete the second color part for the binary pattern. Figure 2e shows the PL images with green and red bandpass filters, and no spatial overlap was found between the green and red areas, indicating that there is no undesired cross-contamination between the two separate printing cycles. This can be further confirmed with micro-area PL spectra (Fig. 2f), in which every pixel only exhibits a single PL peak.

Combinatorial patterning is rare, and it has been only limited to the assembly of two types of NPs in conventional electric field-assisted assembly processes^26,27^. More types of NPs may be assembled on the same substrate by successive assembly cycles, but it usually causes the damage or pollution of the previous assemblies. To further demonstrate the capability of combinatorial patterning, we printed a pseudo-color image with four different NPs. Here15-nm Fe_2_O_3_ NPs were used to create the white color, and NaYF_4_:Yb,Er, CsPbBr_3_ and CdSe@ZnS NPs were used to produce the magenta, green and red colors in the image captured by the dark-field microscopy (Fig. 2g). The images in each printing cycle are shown in Supplementary Fig. 4. Again, no cross-contamination is observed in each pixel, further confirming the robustness of EFASP for printing multiple functional NPs.

This work represents the large-scale deterministic assembly of NPs with multiple types of compositions and functionalities. Although persistent endeavors have been made to achieve high-quality multi-step NP assembly, the previous works failed majorly because the nonspecific adsorption accumulates the assembly errors, and the later cycles inevitably damage the previously assembled structures. In EFASP, however, the error accumulation problem is minimized because the error ratio is extremely low (<2 × 10^-6^) in a single step; and the strong surface adsorption ensures a high endurance of the printed structures to the successive assembly cycles.

### Electrostatic potential and forces

The high performance of EFASP is the result of joint forces of long-range electrostatic interactions and short-range adsorption interactions between NPs and substrate. To have a quantitative understanding of the process, we measured the interaction strengths and calculated how they influence the detailed performance of EFASP.

**Fig. 3:**
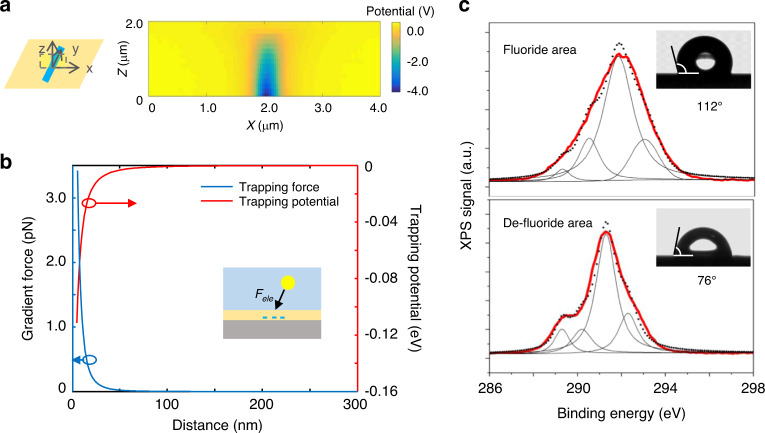
Quantitative analysis of NP–substrate interactions. **a** Electrostatic potential distribution along the x-z plane induced by a charged line with 10 μm length (y axis) and 50 nm width (*x* axis), which is written by applying a voltage of -90 V on the substrate. The electric field is highly localized in the vicinity of the charge line, inducing strong attraction to the surrounding NPs. **b** The calculated electrostatic force and potential of the 1D charged pattern to NPs in a nonpolar solvent, based on the measured potential distribution (**a**). Inset is the schematic illustration of the electrostatic attraction force between the charged pattern and dispersed NPs. **c** XPS spectral analysis of carbon 1 s on charged (de-fluorinated) and uncharged (fluoride) area. The original spectra are shown by the black dotted curve, which is well fitted by the sum (red solid curves) of four Gaussian lines (solid black curves). The high voltage leads to a decrease in the peak at 290.5 and 291.8 eV, which can be assigned as difluoride carbons, and an increase in the peak at 289.3 and 293.0 eV, which can be assigned as the carboxylation carbons. Insets are the corresponding water contact angle of fluoride and de-fluoride area, decreasing from 112^o^ to 76^o^.

To determine the strength of electrostatic interactions, we first wrote a charged line of 10 μm by applying -90 V on the AFM tip, and then mapped its electrostatic potential distribution using the KPFM. As shown in Fig. 3a, the fields were strongly localized in the vicinity of the charge line, causing electrostatic forces, *F*_ele_ on the surrounding NPs.

We can retrieve the charge density of the 1D pattern using the measured potential distribution, and further calculate the electrostatic forces on the NPs in a given solution^28^ (see Supplementary Information Calculation). In our system, the charge density is typically at ~ 1 C/m^2^ level. The *F*_ele_ is the sum of the Coulomb forces, *F*_Coul_, and gradient forces (dielectrophoretic forces), *F*_gra_. Since nonpolar solvents are used and NPs are neutral, Coulomb forces are weak, and the trapping potential is mainly determined by gradient forces. For the case of perovskite QDs in a nonpolar solvent, the trapping potential is approximately 0.1 eV, and trapping forces is over 1 pico-Newton level at the surface (Fig. 3b).

The usage of nonpolar solvent in this work brings several advantages comparing with the case of a commonly used polar solution, particularly water. First, it avoids the screening effects, and the electric fields can extend for hundreds of nanometers, leading to a high collection efficiency of NPs. On the contrary, the electric fields only extend for tens of nanometers in water (when ionic strength is 10^-3^ M) due to the screening effect^29^. Secondly, the dielectric constant of water (as well as other polar solution) is larger than that of NPs. This makes gradient forces repulsive and decreases the adsorption efficiency of NPs further. Third, this process enables access to many high-quality nanoparticles, such as semiconductor quantum dots as most of them are synthesized in nonpolar solvents at elevated temperatures via hot injection or solvothermal processes.

### Surface modification and short-range interactions

Compared with the electrostatic interactions, the short-range interaction induced by surface modification is more complicated. To confirm the chemical modification and consequent surface energy increases of the patterned area, several characterizations before and after charge writing were performed, including X-ray photoelectron spectroscopy (XPS), lateral force measurements, and contact angle measurements.

The carbon 1 s XPS spectra of the substrate surface were shown in Fig. 3c. Four peaks at 290.5, 291.9 289.3, and 293.0 eV were analyzed by Gaussian fitting, which can be assigned to difluoride carbons and de-fluorinated chemical species, respectively. The intensity decrease of the peaks at 290.5 and 291.8 eV, together with the increase at 289.3 and 293.0 eV, indicate the occurrence of de-fluorination induced by the high-voltage writing process (Supplementary Fig. 5). The de-fluorination process can introduce strong modulation of local surface energy, which consequently alters the local frictions. This change in the local friction force was indeed observed in our experiment with lateral force measurement (Supplementary Fig. 6).

The surface energy modulation induced by de-fluorination is significant. As shown in the insets of Fig. 3c, the contact angle with water is changed considerably (from 112 degrees to 76 degrees) by the de-fluorination process. With the help of the combining relation^29^, we can estimate the adhesion energy difference of a 10 nm spherical particle with the substrate before and after it is de-fluorinated, ΔW = 0.24 eV (see Supplementary Information Calculation). In the case of a cubic NP, this value can be even larger. Overall, the adhesion resulting from the change in surface energy is much larger than the electrostatic interaction and provides a strong binding force for the NPs.

### Spatial distribution of NPs

We can further calculate adsorption possibilities of NPs at different areas using Boltzmann-Gibbs distribution

1 $$P\left( {\mathbf{r}} \right) \propto e^{ - U\left( {\mathbf{r}} \right)/k_{\mathrm{B}}T}$$

Here, *P* is the possibility of a nanoparticle being at location **r**, and *U* is the adsorption energy. From Fig. 1c, we learn that the adsorption energy at the patterned area (de-fluorinated area), electrostatic interaction area, and fluoride surface is *U*_defluo_ + *U*_ele_ − *U*_spin_, *U*_ele_ + *U*_fluo_ − *U*_spin_, and *U*_fluo_ − *U*_spin_, respectively. Consequently, the possibility for a nanoparticle to adsorb on the surface is

2 $$P_{{\mathrm{defluo}}} \propto e^{ - \left( {U_{{\mathrm{defluo}}} + U_{{\mathrm{ele}}} - U_{{\mathrm{spin}}}} \right)/k_{\mathrm{B}}T}$$

3 $$P_{{\mathrm{ele}}} \propto e^{ - \left( {U_{{\mathrm{ele}}} + U_{{\mathrm{fluo}}} - U_{{\mathrm{spin}}}} \right)/k_{\mathrm{B}}T}$$

4 $$P_{{\mathrm{fluo}}} \propto e^{ - \left( {U_{{\mathrm{fluo}}} - U_{{\mathrm{spin}}}} \right)/k_{\mathrm{B}}T}$$

Based on this, some of the key parameters of EFASP can be evaluated in a quantitative fashion.

One of the key parameters for the assembly-based nanofabrication technique is the error ratio, which determines whether the technique is useful in industrial settings. In the EFASP process, it can be defined as the ratio between the adsorption possibility in the unpatterned fluoride area and patterned de-fluorinated area,

5 $${\mathrm{ER}} = P_{{\mathrm{fluo}}}/P_{{\mathrm{defluo}}} \propto e^{ - \left( {U_{{\mathrm{defluo}}} + U_{{\mathrm{ele}}} - U_{{\mathrm{fluo}}}} \right)/k_{\mathrm{B}}T}$$

We can see that the error ratio is solely decided by the attraction potential, $\Delta {\mathrm{U}} = U_{{\mathrm{defluo}}} + U_{{\mathrm{ele}}} - U_{{\mathrm{fluo}}}$. Estimation shows that ΔU ~0.35 eV for a 10 nm QD (see Supplementary Information Calculation), and the corresponding error ratio is about 9 × 10^−7^. This explains the extremely clean background over a large area of printed nanostructures demonstrated in Fig. 1d.

### High spatial accuracy

**Fig. 4:**
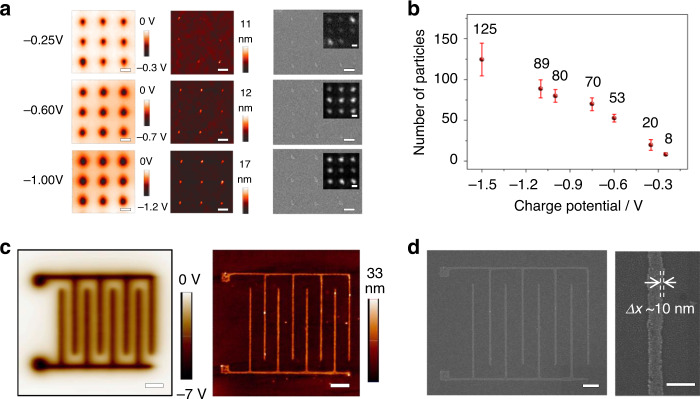
Tunable pixel size and printed interdigitated nanoelectrodes. **a** Tuning the pixel size (the number of NPs on each spot) by adjusting the charge potential. From left to right, they are KPFM potential mapping, corresponding AFM height mapping, SEM, and PL images (inset) of CsPbBr_3_ QD patterns. Scale bar, 500 nm. **b** The near-linear relationship between the average number of NPs on each spot and the charge potential. Error bars represent the standard deviation of the NPs numbers on 100 spots of each potential. **c** KPFM potential image and AFM height mapping of interdigitated nanoelectrode printed with 10-nm Au NPs. Scale bar, 1 μm. **d** Corresponding SEM of the interdigitated nanoelectrode and an enlarged part, showing a finger width of 150 nm with variation less than 10 nm. Scale bar, 1 μm (left), and 200 nm (right).

Another important parameter is the accuracy of the EFASP method. Ideally, all NPs should be limited in the de-fluorinated area. However, from Fig. 1b, we can see that it is possible for NPs to be adsorbed in the nearby area through electrostatic interaction, deteriorating the position accuracy of the EFASP method. Similar to the case of error ratio estimation, we can estimate this uncertainty

6 $$\delta = P_{{\mathrm{ele}}}/P_{{\mathrm{defluo}}} \propto e^{ - \left( {U_{{\mathrm{defluo}}} - U_{{\mathrm{fluo}}}} \right)/k_{\mathrm{B}}T}$$

which is decided by the surface energy modulation depth, $U_{{\mathrm{defluo}}} - U_{{\mathrm{fluo}}}$, caused by the de-fluorination. For a 10 nm spherical NPs, ΔU is around 0.24 eV, and this ratio is 7 × 10^−5^. This leads to a clean-cut between the modified and unmodified area, which is also demonstrated in the printed interdigitated nanoelectrode by Au NPs, where variation less than 10 nm can be achieved for patterns with a line width less than 150 nm (Fig. 4c, d).

### Tunable pixel size

The pixel size in EFASP can be tuned precisely by controlling the number of NPs adsorbed in the patterned area,

7 $$N \propto e^{ - \left( {U_{\mathrm{defluo}} + U_{\mathrm{ele}} - U_{\mathrm{spin}}} \right)/k_BT}$$

To demonstrate this, we fixed the parameters of spin-coating and adjusting *U*_ele_ (0.15 to 1.5 V) by changing the writing voltage applied on the AFM from -40 to -90 V (Supplementary Fig. 7). It is evident that high surface potential leads to a large pixel size (Fig. 4a, b). For a quantitative understanding, the particle volume in the AFM topographic graphs and fluorescence intensity in the PL images were statistically analyzed. All the 100 printed pixels in the 10 × 10 charged spots separated by 1.0 μm were counted, as shown in Supplementary Fig. 8. Both the two plots (Supplementary Fig. 8e, f) demonstrate that there is a near-linear relationship between the average particle number and the surface potential.

We can push the pixel size down to few-NP level by lowering the pattern writing potential to -0.15 V. Only several NPs are adsorbed in one pixel, while still maintaining 100% yield based on the charged spots. Taking 15-nm Fe_2_O_3_ NPs as an example, only 2–5 NPs are assembled at each spot when the surface potential is low at -0.25 V (Supplementary Fig. 10c), corresponding to a pixel size less than 30 nm. This few-NP level control capability is particularly useful for making QD nanopatterns since single QDs can be used for generating single photons, which have many applications in the field of quantum information. For illustrating this, a 10 × 10 array of CsPbBr_3_ QDs was assembled at the surface potential of -0.15 V, and low-temperature PL spectra (at 4 K) show that there are only a few well-separated fluorescence peaks at each pixel (Supplementary Fig. 9), indicating that QDs can be precisely located at the desired position at the few-NP level since one CsPbBr_3_ QD normally only has one emission peak at low temperature.

### Printing continuous nanostructured assemblies

In addition to the few-NP level pixel control, the EFASP technique is suitable for fabricating continuous nanostructured assemblies, which are particularly useful in electronics, e.g., conductive nanowires, antennas, as well as nano-optoelectronic devices. As an example, we prepared interdigitated nanoelectrodes printed using Au NP (10 nm, Fig. 4c, d) with 1 μm electrode separation and <150 nm finger width. Note that nonpolar solvents are used in this work and NPs are neutral, and therefore, the NPs are densely packed when they are assembled on the patterns. This is different from the water-dispersed NPs which are repulsive to each other due to the existence of surface charges.

### Versatility of the patterning technique

The EFASP technique is versatile for various nanoparticle inks, insensitive to printing parameters, and easy to scale up. As a physical adsorption process, the EFASP technique is not sensitive to the chemical properties of the ink materials, and therefore, all synthesized functional NPs dispersed in a nonpolar solvent can be used as the ink for effective nanoscale printing. We tested several different NPs including magnetic, metal, semiconductor, dielectric nanomaterial (e.g., γ-Fe_2_O_3_, Au, CdSe, and NaYF_4_, respectively) with the size from a few nanometers to tens of nanometer, and high quality printed structure can be obtained for all of these materials without exception (Supplementary Fig. 10).

Finally, it is worth noting that there is no limit on the printing size of the EFASP technique. Currently, the size is only limited by the nanopattern writing tool (i.e., the AFM), and this potential problem can be solved by many existing techniques, e.g., tip arrays or a large area mold^30,31^. In addition, we can set the scan AFM head on the motorized stage to address the concerns about the slow and small scale about AFM based technology. As demonstrated in Supplementary Fig. 11, by combining the tip scanning and stage moving, we can easily make millimeter-scale charge patterns in 15 min, requiring no manual tip-repositioning step.

## Discussion

In this work, we have developed a nanofabrication technique, EFASP, which is efficient, fast, robust, accurate, and widely applicable. By regulating the long-range and short-range forces between the oil-dispersed NPs and the substrate, as well as the surface energy of the substrate, which can be achieved by high-voltage tip-based writing, we can print the NPs into nanopatterns with feature size less than 30 nm, without any nonspecific adsorption. The EFASP is versatile for NPs dispersed in nonpolar solvents, such as QDs, magnetic NPs, upconversion fluorescent nanocrystals and metal NPs. Moreover, we have demonstrated that multiple NPs could be assembled accurately on the same substrate with an arbitrary arrangement, just by repeating successive charge-writing/spin-coating cycles. The printing process is intrinsically scalable, and samples over millimeter sizes can be produced if the high-voltage writing is carried out using tip arrays or large-area molds. This work provides a platform for convenient, flexible, and rapid manufacturing of non-silicon based nanodevices, such as integrated photonic chips, biochips, and wearable devices.

## Methods

### Synthesis

All the perovskite QDs were synthesized by the hot injection method^32^. Take the typical perovskite CsPbBr_3_ QDs as an example, Cs_2_CO_3_ (0.407 g) was loaded into a 50 ml three-neck flask with octadecene (ODE, 20 ml) and oleic acid (1.5 ml). The mixture was degassed for 0.5 h at 120 °C, and then heated to 150 °C under Ar to dissolve the solid completely, as the Cs-oleate stock solution. In another three-neck flask, PbBr_2_ (0.14 g) was added into a mixed solvent of ODE (10 ml), oleylamine (2 ml), and oleic acid (1 ml). The mixture was then heated to 120 °C under vacuum, followed by Ar gas flow. After complete solubilization of the PbBr_2_ salt, the temperature was raised to 150 °C, and followed by a quick injection of above Cs-oleate solution (1 ml). The reaction was stopped after 5 s by an ice-water bath. After the dispersion of perovskite NCs was cooled down to 30 °C, antisolvent such as acetone with volume ratio 1:1 to original dispersion was added to precipitate NCs. The resulting CsPbBr_3_ NCs were collected by centrifugation, and then re-dispersed into 3 ml cyclohexane. With the same steps, CsPbI_3_ NCs can be synthesized by replacing the PbBr_2_ salts with the same mole amount of PbI_2_ salts.

Superparamagnetic γ-Fe_2_O_3_ NPs were synthesized using a thermolysis process^33^. Fe(CO)_5_ (0.2 mL, 1.52 mmol) was added to a mixture containing 10 mL of ODE and 1.28 g of oleic acid at 100 °C. The solution was then heated to 290 °C under Ar atmosphere and maintained for 1 h. Then the solution was cooled down to 200 °C and bubbled with air for 2 h. After cooling down to room temperature, acetone was added to precipitate γ-Fe_2_O_3_ nanoparticles. Finally, the resulting black powder was collected by centrifugation and re-dispersed into 10 mL cyclohexane.

Au NPs were synthesized as follows^34^. For the synthesis of 5 nm Au nanoparticles, 0.0925 g dodecyldimethylammonium bromide (DDAB) and 0.046 g HAuCl_4_·4H_2_O were added into 10 mL of toluene with ultrasonication until the complete dissolution of metal salts. In total 40 μL of freshly prepared aqueous NaBH_4_ (9.4 M) solution were added dropwise under vigorously stirring to initiate the gold reduction. After 20 min, 0.8 mL 1-dodecanethiol was added into the as-prepared solution, and the stirring was continued for 5 minutes. After adding ethanol, Au NPs were precipitated and separated through centrifugation. Finally, the resulting Au NPs were re-dispersed into 10 ml cyclohexane.

Upconversion fluorescent NaYF_4_:18%Yb, 2%Er NPs were synthesized as follows^35^. YCl_3_·6H_2_O (242.7 mg), YbCl_3_·6H_2_O (70.0 mg), and ErCl_3_·6H_2_O (7.6 mg) were dissolved in 100 μL water first and then mixed with oleic acid (6 mL) and 1-octadecene (15 mL) in a three-neck flask. The solution was degassed at 150 °C under Ar for 30 min and then cooled down to room temperature. A 10-mL methanol solution containing NaOH (0.1 g) and NH_4_F (0.1481 g) was added and stirred for 30 min. After that, the solution was slowly heated to 110 °C and kept at 110 °C for 0.5 h to remove methanol and a small amount of water. Then, the solution was quickly heated to 320 °C and aged for 1 h under Ar protection. After the solution was cooled down, acetone was added to precipitate the nanoparticles. The final NaYF_4_:Yb,Er NPs was re-dispersed in 5 mL of cyclohexane after washing with cyclohexane/acetone two times.

The CdSe/ZnS core/shell NP dispersions were purchased from Suzhou Xingshuo Nanotech Co., Ltd.

All the TEM images of the functional NPs are shown in Supplementary Fig. 12.

### Charge writing and NP assembly

Charge writing was performed using a customized AFM-based direct electrostatic charge writing system, which contains an atomic force microscope from NT-MDT NTEGRA PROBE NANOLABORATORY mounted with TipsNano HA_NC/Pt tips (235/140 kHz, 12/3.5 N/m, 20-30 nm Pt coating) and an external high voltage amplifier manufactured by THORLABS (model: HVA200). A commercial fluorine polymer CYTOP (perfluoro (1-butenyl vinyl ether)) and its special dilution solvent (perfluorocarbons, PFC) were used as a substrate (purchased from ASAHI GLASS COMPANY, Japan). Typically, CYTOP powder was diluted to 1–3 wt% with solvent and spin-coated onto silicon wafers, with spin speed, spin time, and acceleration set as 2500 rpm, 1 min, and 1200 r/min^2^, respectively. Then the substrate was baked on a hot plate at 100 ^o^C for 10 min to remove residual solvent. For writing charged patterns on the polymer substrate, the tips were loaded at high voltage and driven using the Litho mode of Nova software. The z-feedback was adjusted to control the tip–substrate distance during charge writing. In order to import a preset pattern, Vector mode was selected to write the simple dot charge matrix, and Raster mode was used to write the complex charge patterns. The time on a single point was fixed at 100 ms and 18 ms for Vector and Raster mode, respectively. The electrical voltage added on tips is -40 to -90V, which can generate -0.15 to -1.5 V charge potential on the substrate. Charge writing was performed in the air under ambient conditions (room temperature, relative humidity = 20–40%).

After charge pattern writing, the nanoparticles were printed by spin-coating of diluted colloidal nanoparticle suspension. The typical spin speed, spin time, and acceleration were set as 6000 rpm, 25 s, and 1200 r/min^2^, respectively. The typical dispersion concentration was 1.5 × 10^13^ NPs/mL. As long as the dispersion covered the charge pattern, NPs could be successfully assembled onto the designated area. For Supplementary Movie 1, we just put one drop of the NP dispersion (10 μL) next to the pattern, and then use a needle to swipe the drop through the patterns (around 0.5 cm/s). The NPs can be assembled on the patterns quickly and efficiently. All the operations can be performed in the open system, generally in the fume hood at RT, including spin-coating, dip coating, or brushing.

### Measurement and characterization

All the AFM-based experiments were carried on an INTEGRA SPM Controller of NT-MDT. After AFM charge writing, the surface potential of the charge patterns (relative to the surrounding uncharged polymer substrate) was measured by Kelvin Probe Force Microscopy (KPFM) in semi-contact mode. In the KPFM experiments, double path measurement was used, and the typical lift height was 10 nm. After NP assembling, the height images were obtained by tapping mode scanning (carrying an NSG10 tip of TipsNano).

The fluorescence images of quantum dot patterns were obtained using a fluorescence microscope (Newton EMCCD, ANDOR) with a 405-nm laser. The PL spectrum was measured using an SR-303i-B spectrograph of Andor (EU). The single point FL spectrums of low temperature were measured by Andor spectrograph at 4 K.

The SEM images were captured using a Zeiss Ultra55 field-emission SEM operated at 15 kV. X-ray photoelectron spectroscopy (XPS, K-Alpha, Thermo Scientific, UK) were in situ measured after writing dense charge lattice with 200 × 200 μm area. The TEM images were captured by FEI Tecnai F20 G2 TEM.

## Supplementary information

Supplementary Information

Description of Additional Supplementary Files

Supplementary Movie 1

## Data Availability

The data that support the findings of this study are available from the corresponding author upon reasonable request.
