# Supplementary material for: High-resolution combinatorial patterning of functional nanoparticles
Source: Nat Commun. 2020 Nov 26;11:6002. doi: 10.1038/s41467-020-19771-0 (PMC7691364; doi:10.1038/s41467-020-19771-0)
Supplement: Supplementary file 1 — Supplementary Information [file 41467_2020_19771_MOESM1_ESM.pdf]

Supplementary Information

High-Resolution Combinatorial Patterning of Functional Nanoparticles

Xing Xing[1,†], Zaiqin Man[1,†], Jie Bian[1,†], Yadong Yin[2*], Weihua Zhang[1,3*] & Zhenda Lu[1,4*]

[1]College of Engineering and Applied Sciences, State Key Laboratory of Analytical Chemistry for Life Science, and Jiangsu Key Laboratory of Artificial Functional Materials, Nanjing University, Nanjing 210093, P. R. China

[2]Department of Chemistry, University of California, Riverside, California 92521, USA.

[3]MOE Key laboratory of Intelligent Optical Sensing and Manipulation, Nanjing University, Nanjing 210023, P. R. China.

[4]Research Center for Environmental Nanotechnology (ReCENT), Nanjing University, Nanjing 210023, P. R. China.

[†]These authors contributed equally: Xing Xing, Zaiqin Man, Jie Bian

*E-mail: yadong.yin@ucr.edu; zwh@nju.edu.cn; luzhenda@nju.edu.cn

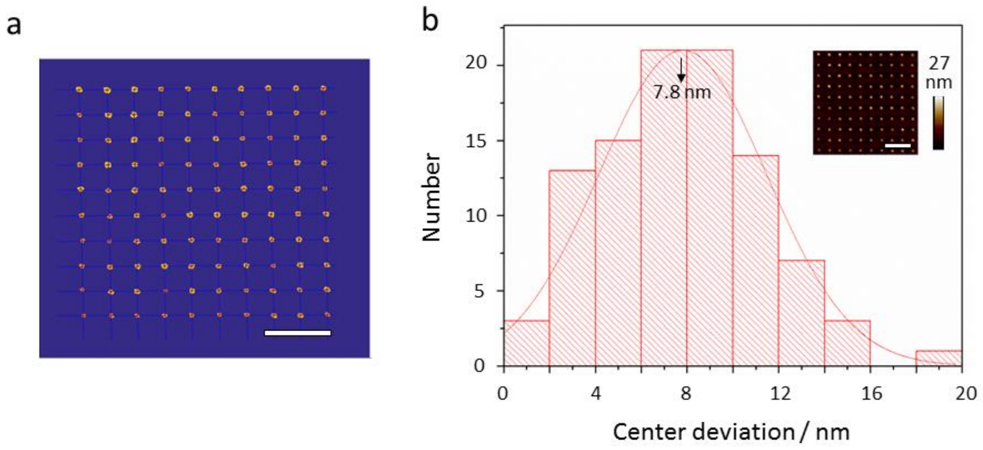

Supplementary Fig. 1| Position accuracy is better than 10 nm. The assembly accuracy is determined by the following method. First, the assembly area is determined by the AFM height image of NP patterns, and the center of each spot represents the experimental position of the printing. The theoretical assembly position is determined by the 10 × 10 array with a known spacing (corresponds to the periodic spacing of the potential matrix), scale bar: 1 μm. The position accuracy is then calculated as the positional deviation between the experimental center and the theoretical center of each spot. Finally, 100 deviation values were analyzed by statistical histograms. Take the 10 × 10 dot matrix at 400 nm spacing as an example, the AFM height image, fitting result (Fig. a) and statistical result (Fig. b) are shown, and the average center deviation is 7.8 nm.

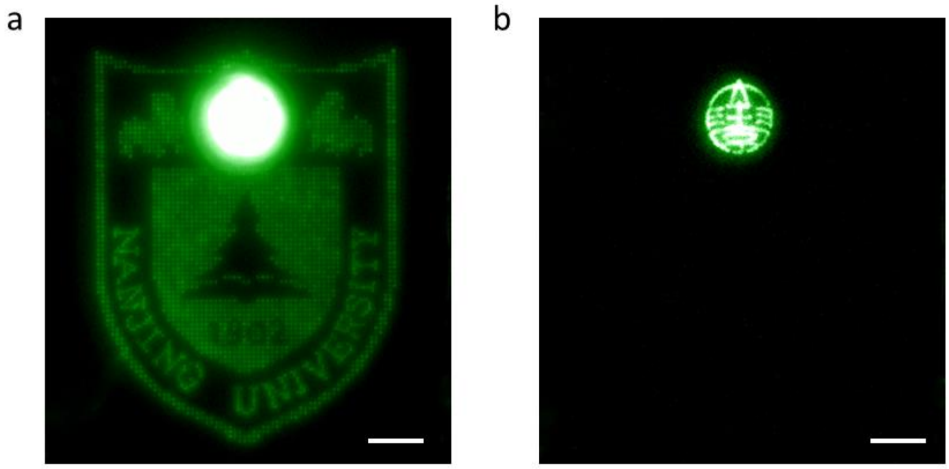

Supplementary Fig. 2| PL image of Nanjing University Badge fabricated with different pitches. The center circular mark has dense pixels, reaching 200 nm pitch, while the other area keeps 800 nm pitch. The two PL images were taken in the same area with a different exposure time of 20 ms (a) and 1 ms (b), respectively. Scale bar: 10 μm.

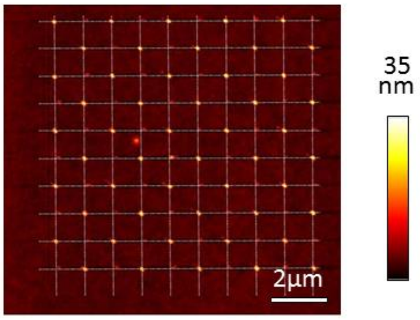

Supplementary Fig. 3| Overlay accuracy between different cycles reaches 58 nm. The first printing cycle (brighter spots) is assembled by 13 nm CdSe NPs in 5 × 5 array with 2 μm spacing, and then the second array is assembled in the intervals (darker sport). The two print cycles form a 10 × 10 dot matrix with a pitch of 1 μm. Based on the first cycle, the white dashed grid in the figure is fitted by the center of each spot in the first layer. The intersections in the grid are taken as the theoretical centers of the second printing cycle, and its average deviation between the theoretical center and the actual center of each spot is 58 nm.

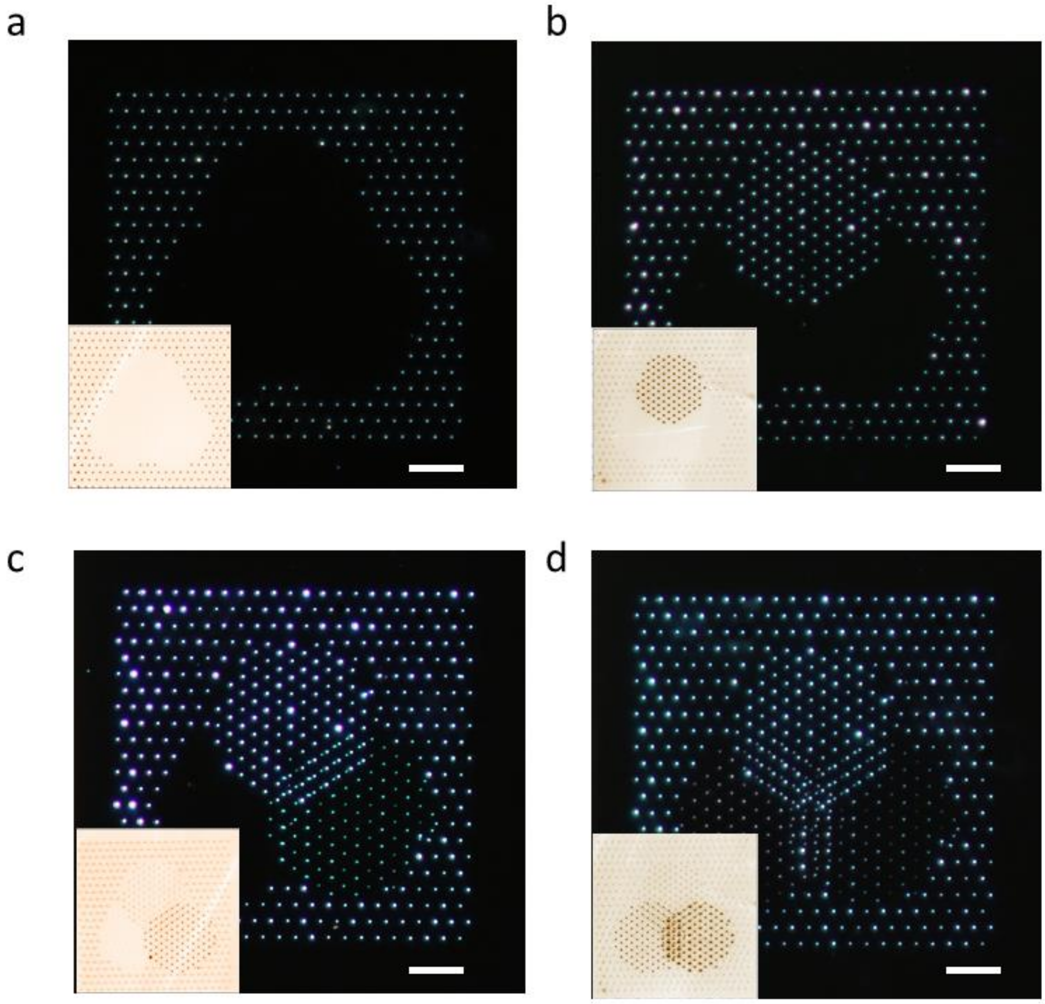

Supplementary Fig. 4| Dark field images of each step in the four-cycle overlay procedures. a, The first cycle assembled with γ-Fe_2O_3 nanoparticles. b, The second cycle assembled with $NaYF_4$:Yb,Er NPs. c, The third cycle assembled with $CsPbBr_3$ NPs. d, The fourth cycle assembled with red CdSe/ZnS QDs (620 nm). Scale bar: 10μm. The KPFM potential images in the insets show the surface potentials at the corresponding cycles.

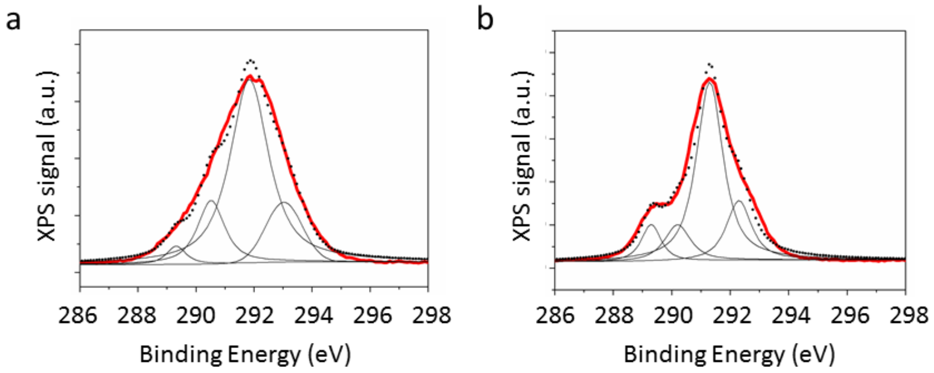

c

Binding Energy (ev)	289.28	290.48	291.85	293.05
	MeCOOH	$(CF_2CH_2)_n$	$(CF_2)_n$	CF_3COOEt
Before	3.32%	16.04%	66.02%	14.62%
After	9.23%	12.09%	59.95%	18.73%
	↑	↓	↓	↑

Supplementary Fig. 5| The carbon 1s XPS analyses of charged and uncharged areas. a, Uncharged area. b, charged area. The original spectra are shown by black dotted curves, which are well fitted by the sum (red solid curves) of four Gaussian lines (solid black curves for each), with peaks at 289.28, 290.48, 291.85 and 293.05 eV, respectively. c, The table showing the high voltage leads to a decrease in the peaks at 290.48 and 291.85 eV, while an increase in the peaks at 289.28 and 293.05 eV, which can be assigned to fluoride carbons and carboxylation carbons, respectively.

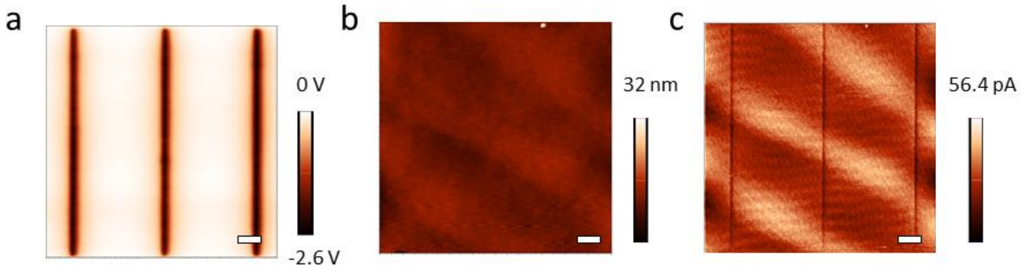

Supplementary Fig. 6| Lateral force mapping of the charged area. a, KPFM potential image of three lines with the surface potential of -2.5 V. b, c, Its corresponding height image (b) and lateral force image (c) in the same area as Fig. a. Scale bar, 2μm.

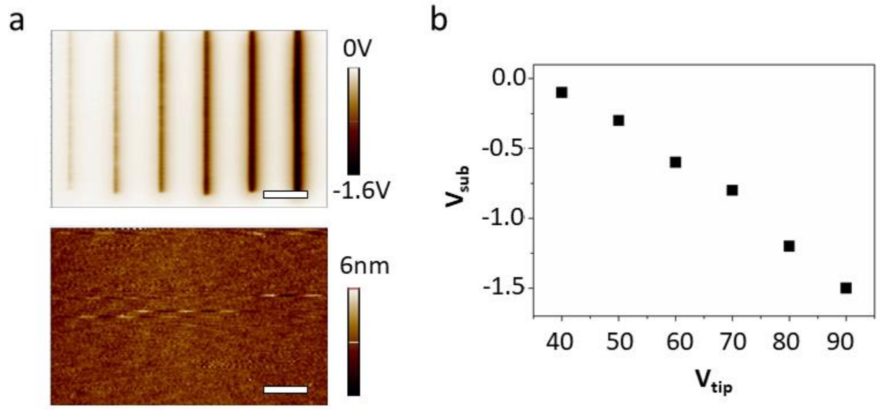

Supplementary Fig. 7| Adjusting surface potential by changing the writing voltage applied on the AFM tip. a, KPFM potential image and its corresponding height image of six charged lines applied different voltage using an AFM tip. It shows that charge writing has no effect on the surface topology of the substrate. Scale bar, 5μm. b, The nearly linear relationship between the surface potential on the substrate and the voltage applied to the tip.

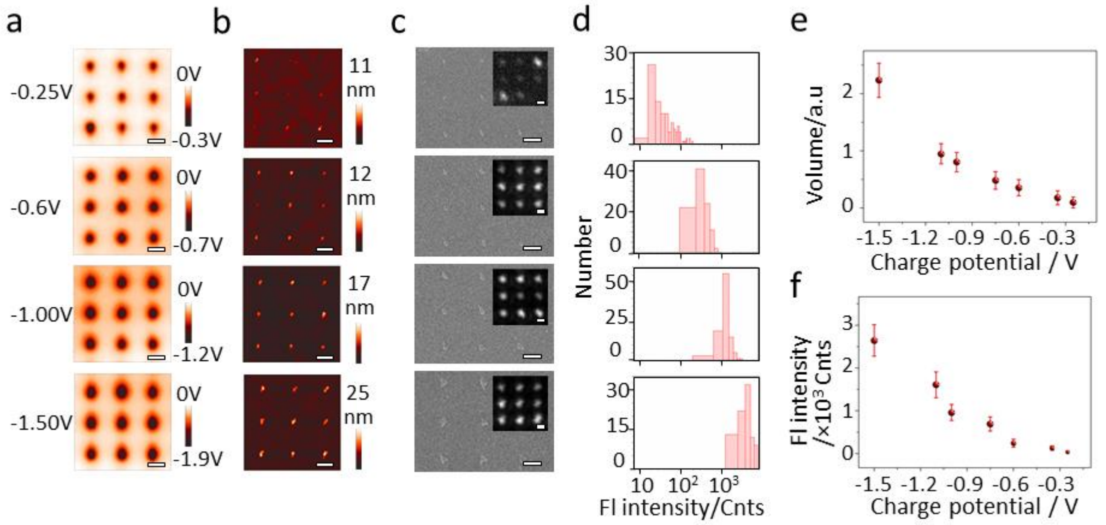

Supplementary Fig. 8| Pixel size control by tuning the surface potentials. a, KPFM potential images. b, AFM height images. c, SEM and FL microscope (inset) images. Scale bar, 500nm. d, Statistical distribution diagram of fluorescence intensity at different potentials. 100 dots were counted for each potential. e, Relationship between particle volume at a single dot (determined from AFM topology) and charge potential. f, Relationship between FL intensity at single dot and charge potential. Error bars represent the standard deviation of the volume or Fl intensity on 100 spots of each potential.

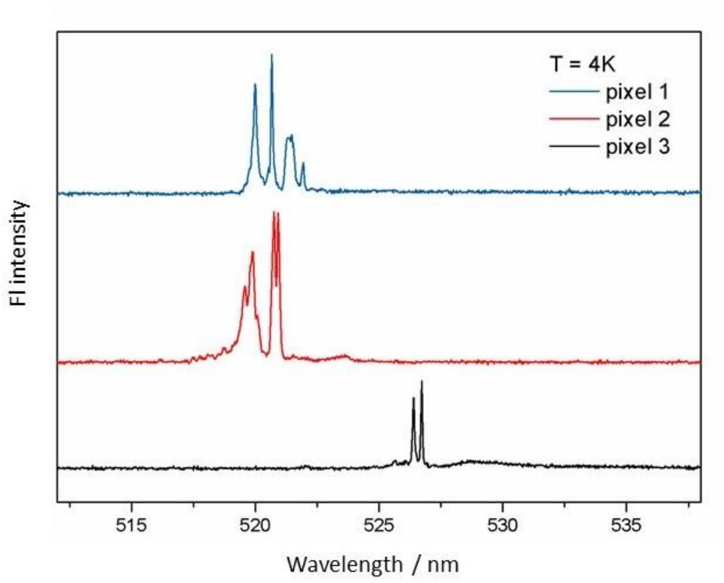

Supplementary Fig. 9| low-temperature PL spectra (at 4K) at each pixel of $CsPbBr_3$ QD assembly. It indicates that there are only a few QDs on each pixel since one $CsPbBr_3$ QD only has one emission peak at a low temperature.

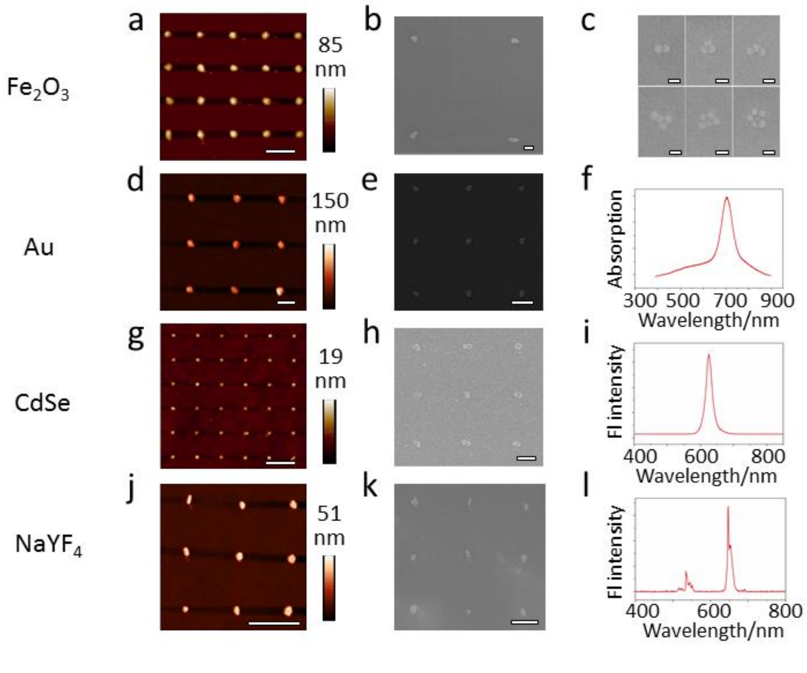

Supplementary Fig. 10| Universality of the EFASP method for different functional NPs. AFM topology mapping, SEM image and corresponding optical properties of patterns assembled by different kinds of NPs. a-c, magnetic NPs (15 nm Fe_2O_3). d-f, metal NPs (5 nm Au). g-i, semiconductor QDs (14nm CdSe/ZnS core/shell NPs with an emission peak at 620 nm). j-l, Upconversion fluorescence NPs (22 nm $NaYF_4$: Yb,Er NPs). Scale bar, 1μm for Fig a, d, g and j. 100 nm, 1um, 200 nm and 500 nm for Fig b, e, h and k. 30 nm for Fig c.

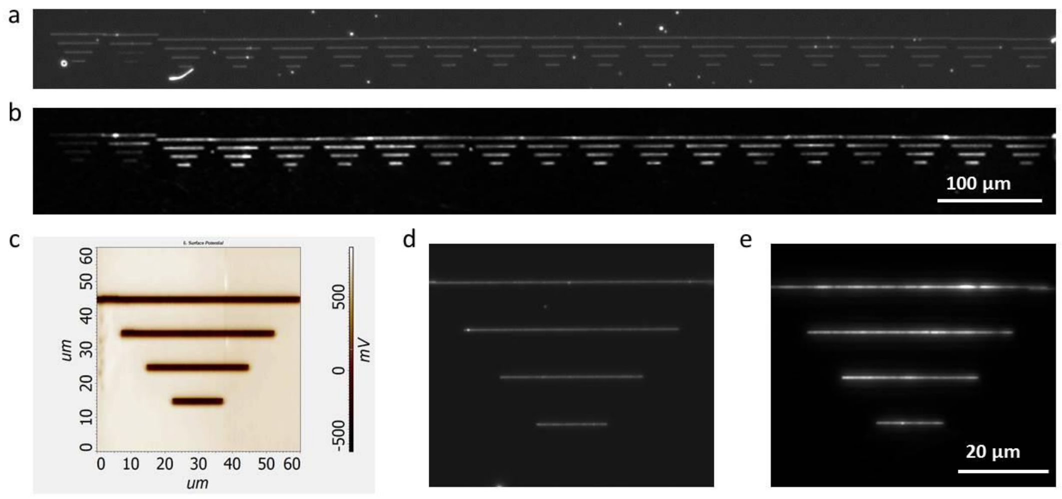

Supplementary Fig. 11| Nanoprinting of perovskite QDs in millimeter scale. a-b, A large-scale dark-field microscopy image and photoluminescence (PL) image obtained by assembling $CsPbBr_3$ NPs using the EFASP process, with a total area of 0.06 × 1.2 mm^2. c, The KPFM potential map of the unit pattern of four different lines in the area of 60 × 60 μm^2. d-e, The high-magnification dark-field microscopy image (d) and the PL image of NP assembly (e) corresponding to the unit pattern.

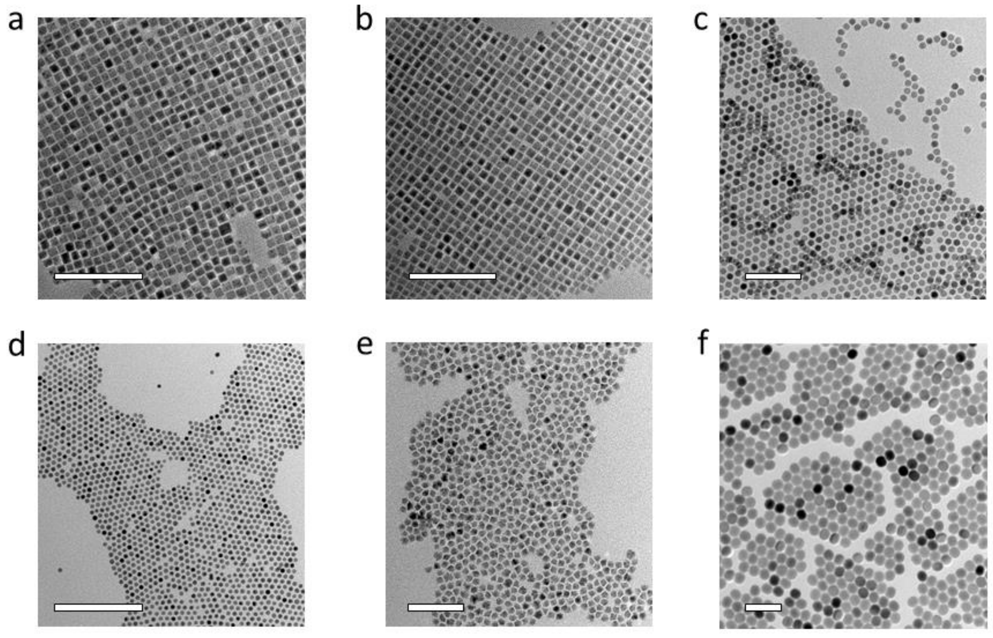

Supplementary Fig. 12| TEM images of different functional NPs. a, $CsPbBr_3$ NPs. b, $CsPbI_3$ NPs. c, γ-Fe_3O_4 NPs. d, Au NPs. e, CdSe/ZnS core/shell NPs. f, $NaYF_4$/Yb, Er NPs. Scale bar, 100nm.

Calculation

charge induced electric field and forces

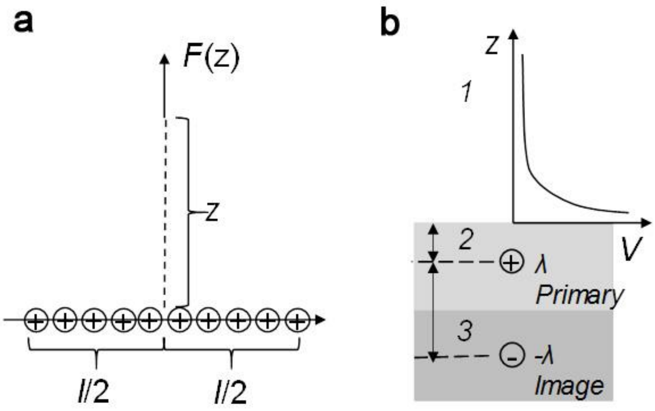

Supplementary Fig. 13| a, Electric field induced by a line charge was measured along the central axis by Kelvin probe force microscopy. b, Field in media 1 is caused by both the primary line charge in media 2 and image line charge in 3 (conductor).

The charge density in the patterned area can be retrieved using the electrical field potential distribution measured by Kelvin probe force microscopy. Here, we mapped the potential field of a 10 μm line charge pattern at different height z along the symmetrical axis, as shown in Supplementary Fig. 13a. For simplicity, we use an ideal line charge model (with line charge density, λ_1) to describe the system. Since the substrate 3 is conductor, there is an additional image charge line with opposite signs inside the substrate (line charge density λ_2), as shown in Supplementary Fig. 13b.

In free space, the electric potential induced by a charge line (Supplementary Fig. 13a) is:

$$V = \frac{1}{4\pi\varepsilon_0}\lambda \ln\left(\frac{\frac{l}{2}+\sqrt{\left(\frac{l}{2}\right)^2+z^2}}{-\frac{l}{2}+\sqrt{\left(\frac{l}{2}\right)^2+z^2}}\right) \tag{s1}$$

Then, the total potential by both the primary charge line and image charge line becomes:

$$V = \frac{1}{4\pi\varepsilon_0}\lambda_1 \ln\left(\frac{\frac{l}{2}+\sqrt{\left(\frac{l}{2}\right)^2+(z+d_1)^2}}{-\frac{l}{2}+\sqrt{\left(\frac{l}{2}\right)^2+(z+d_1)^2}}\right) + k\lambda_2 \ln\left(\frac{\frac{l}{2}+\sqrt{\left(\frac{l}{2}\right)^2+(z+d_2)^2}}{-\frac{l}{2}+\sqrt{\left(\frac{l}{2}\right)^2+(z+d_2)^2}}\right) \tag{s2}$$

Finally, since the charges are embedded in the matrix 2, there is an additional factor $\frac{2\varepsilon_2}{\varepsilon_1+\varepsilon_2}$ for the potential measured in media 1. Therefore, we have

$$V = \frac{1}{4\pi\varepsilon_0}\frac{2\varepsilon_2}{\varepsilon_1+\varepsilon_2}\lambda_1 \ln\left(\frac{\frac{l}{2}+\sqrt{\left(\frac{l}{2}\right)^2+(z+d_1)^2}}{-\frac{l}{2}+\sqrt{\left(\frac{l}{2}\right)^2+(z+d_1)^2}}\right) + k\lambda_2 \ln\left(\frac{\frac{l}{2}+\sqrt{\left(\frac{l}{2}\right)^2+(z+d_2)^2}}{-\frac{l}{2}+\sqrt{\left(\frac{l}{2}\right)^2+(z+d_2)^2}}\right) \tag{s3}$$

The measured data can be fitted with eq. s3 almost perfectly, as shown in Supplementary Fig. 14. The result shows that line charge is 7.7×10^{-11} C/m, 6 nm below the substrate/air interface. Assuming that the line width is 30 nm, the area density of charge is 2.6 C/m^2, which is consistent with other reports[1].

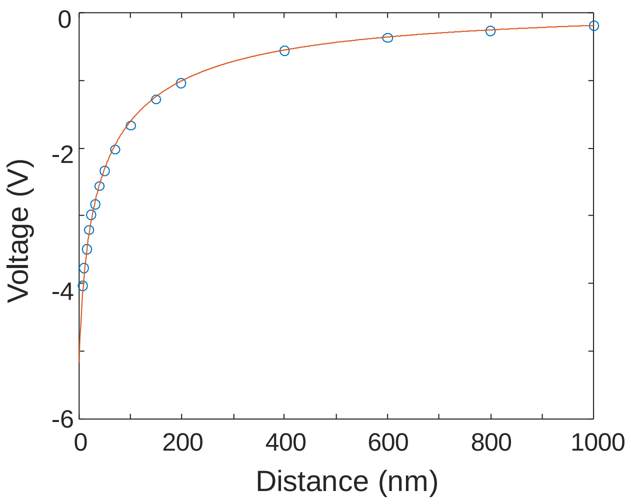

Supplementary Fig. 14| Voltage-distance curve. The hollow dots are experimental data, and the solid line is the fitting result.

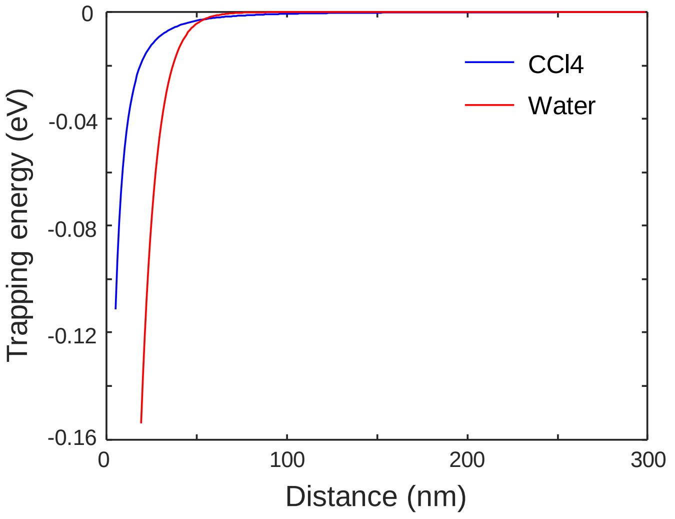

Supplementary Fig. 15| Electric field-induced trapping potential at different distances from the substrate/solution interface.

With the charge distribution data, the field-induced trapping potential and forces can be calculated. In the case of a nonpolar solution, the forces are caused by the field gradient (i.e., dielectrophoretic forces).

$$\mathbf{F}_{dep} = 2\pi\varepsilon_0\varepsilon_1 Re\left(\frac{\varepsilon_1-\varepsilon_{NP}}{\varepsilon_1+2\varepsilon_{NP}}\right)\nabla|\mathbf{E}|^2 \quad \text{(s4)}$$

Then, we have

$$U_{dep} = \int_{\infty}^{z} F_{dep}(z')dz' = 2\pi\varepsilon_0\varepsilon_1 Re\left(\frac{\varepsilon_1-\varepsilon_{NP}}{\varepsilon_1+2\varepsilon_{NP}}\right)|\mathbf{E}|^2 \quad \text{(s5)}$$

In the case of a polar solution, for instance, water, NPs carry net charges, and therefore the electrophoretic forces dominate.

$$\mathbf{F}_{ep} = Q_{eff}\mathbf{E} \quad \text{(s6)}$$

And the potential is

$U_{ep} = Q_{eff}V$ (s7)

Here, Q_{eff} is the effective charge of the NP.

It is worth emphasizing that the solution has a strong influence on the field distribution. In the case of a nonpolar solution, eq. s3 can be directly used. In the case of water, the presence of ions will cause strong screening effects which lead to an exponential decay of the fields in the solution $e^{-z/K}$. Here, $K = \sqrt{\varepsilon_0 \varepsilon k_B T / 2e^2 \rho}$ is the Debye length, k_B is the Boltzmann constant, and ρ is the ionic strength of the electrolyte.

To estimate the strength of electric interactions, we assume $r = 5$ nm, $Q_{eff} = 10$, and $\varepsilon_{NP} = 6.2$. The potential and forces in nonpolar solution and water can then be calculated directly using eq. s 4-7, as shown in Supplementary Fig. 15. Here, the minimum distance in water is set to $2K$ because of the two screening layer at the solid/water interfaces.

Van de Waals forces between NPs and substrate

In this part, we calculate the difference of binding energies between on the patterned and on the unpatterned area in theory. In the case of spherical particle above a planar substrate, the binding energy is

$\mathrm{W} = -AR/6D$ (s8)

The force is

$\mathrm{F} = -AR/6D^2$ (s9)

Here, A is the Hamaker constant. If we assume our system consists of three different types of media, substrate, nanoparticle and solution, respectively, Hamaker constant becomes

$A_{132} = \left(\sqrt{A_{11}} - \sqrt{A_{33}}\right)\left(\sqrt{A_{22}} - \sqrt{A_{33}}\right)$ (s10)

In this work, the high voltage modified the substrate, and the Hamaker constants changed from A_{11} to $A_{1'1'}$. The energy difference between the patterned (modified) and unpatterned (unmodified) surface becomes

$\Delta\mathrm{W} = W_{1'} - W_1 = \frac{R}{6D}\left(\sqrt{A_{1'1'}} - \sqrt{A_{11}}\right)\left(\sqrt{A_{22}} - \sqrt{A_{33}}\right)$ (s11)

The change of Hamaker constant of the substrate can be calculated from the surface energy, γ_1, using the following relation

$\mathrm{W} = -A/12\pi D_0 = -2\gamma$ (s12)

Here, $D_0 = 0.165$ nm, and surface energy of the solid can be determined by contact angle experiment and Young equation:

$\gamma_{12} + \gamma_2 \cos\theta = \gamma_1$ (s13)

Here, media 2 is liquid (water in our case). It is known that surface energy between solid and liquid follows a simple relation:

$\gamma_{12} \approx \gamma_1 + \gamma_2 - 2\sqrt{\gamma_1\gamma_2}$ (s14)

We then have

$\gamma_1 = \frac{1}{2}\sqrt{\gamma_2}(1 + cos\theta)$ (s15)

Combing eq. s11-s15, we obtain

$\sqrt{A_{1'1'}} - \sqrt{A_{11}} = \sqrt{6\pi}D_0\sqrt{\gamma_2}(cos\theta' - cos\theta)$ (s16)

Then, the binding energy difference between the patterned and unpatterned area becomes

$$\Delta W = \frac{RD_0}{D}\sqrt{\frac{\pi\gamma_2}{6}}(cos\theta' - cos\theta)\left(\sqrt{A_{22}} - \sqrt{A_{33}}\right) \qquad (s17)$$

To calculate the energy change, we set $A_2 = 10\times10^{-20}$ J (CdS), $A_3 = 5.5\times10^{-20}$ J (CCl4), R = 5 nm and D = 0.2 nm (directly contact. We also measured the contact angles between the water (γ = 0.072 N/m) and the substrate. Using eq. s17, we have $\Delta W = 0.2380$ eV

It is worth noting that binding energy is highly sensitive to the geometry of the NP. In the case of a nanocube, the binding energy change is

$$\Delta W = \frac{l^2 D_0}{2D^2}\sqrt{\frac{\gamma_2}{6\pi}}(cos\theta' - cos\theta)\left(\sqrt{A_{22}} - \sqrt{A_{33}}\right) \qquad (s18)$$

This will lead to a binding energy contrast larger than 1 eV. The high binding energy contrast between the patterned and unpatterned area explains the clean background in this work.

Reference

1 Palleau, E., Sangeetha, N. M. & Ressier, L. Quantification of the electrostatic forces involved in the directed assembly of colloidal nanoparticles by AFM nanoxerography. *Nanotechnology* **22**, 325603 (2011).
