# Supplementary material for: High-resolution combinatorial patterning of functional nanoparticles
Source: Nat Commun. 2020 Nov 26;11:6002. doi: 10.1038/s41467-020-19771-0 (PMC7691364; doi:10.1038/s41467-020-19771-0)
Supplement: Supplementary file 2 — Description of Additional Supplementary Files [file 41467_2020_19771_MOESM2_ESM.pdf]

Description of Additional Supplementary Files

File Name: Supplementary Movie 1
Description: The movie shows optical microscope observations of the words “NJU nano printing fast robust precise” in a 120×120 μm^2 area produced by the EFASP method, by brushing the γ-Fe_2O_3 NPs on the charged patterns. The words “NJU Nano printing” is printed in the first cycle and followed by the “fast robust precise” in the second cycle
